# The global change of gene expression pattern caused by PTEN mutation affects the prognosis of glioblastoma

**DOI:** 10.3389/fonc.2022.952521

**Published:** 2022-08-09

**Authors:** Shengjun Zhou, Haifeng Wang, Yi Huang, Yiwen Wu, Zhiqing Lin

**Affiliations:** Department of Neurosurgery, Ningbo City First Hospital, Ningbo, China

**Keywords:** prognosis, LASSO, pan-cancer analysis, GBM, glioblastoma

## Abstract

Glioblastoma (GBM), an aggressive primary tumor, is common in humans, accounting for 12–15% of all intracranial tumors, and has median survival of fewer than 15 months. Since a growing body of evidence suggests that conventional drugs are ineffective against GBM, our goal is to find emerging therapies that play a role in its treatment. This research constructs a risk model to predict the prognosis of GBM patients. A set of genes associated with GBM was taken from a GBM gene data bank, and clinical information on patients with GBM was retrieved from the Cancer Genome Atlas (TCGA) data bank. One-way Cox and Kaplan–Meier analyses were performed to identify genes in relation to prognosis. Groups were classified into high and low expression level of PTEN expression. Prognosis-related genes were further identified, and multi-factor Cox regression analysis was used to build risk score equations for the prognostic model to construct a survival prognostic model. The area under the ROC curve suggested that the pattern had high accuracy. When combined with nomogram analysis, GJB2 was considered an independent predictor of GBM prognosis. This study provides a potential prognostic predictive biological marker for GBM patients and confirms that GJB2 is a key gene for GBM progression.

## Introduction

Glioblastoma (GBM) is an aggressive primary tumor that is the most aggressive and common type of brain tumor in humans (1), with a median survival of fewer than 15 months (2). It consists of highly malignant cells with metastatic and angiogenic properties that lead to resistance to agents such as temozolomide, although none of these agents significantly improves overall survival (3). The conventional treatment is a combination of chemotherapy and surgical resection followed by radiotherapy and adjuvant chemotherapy. This regimen has been effective in improving overall survival but largely fails to prevent recurrence because surgical treatment fails to completely eradicate GBM cells, which remain surrounded by scattered GBM-infiltrating cells (4). Therefore, the identification of reliable prognostic markers has become crucial for GBM treatment.

Phosphatase and tensin homolog (PTEN), a tumor suppressor gene, is closely involved in cell translation, proliferation, and tumorigenesis. PTEN gene mutations are frequently found in the genetic landscape of high-grade gliomas, and is hallmarks of glioma malignancy,they influence cell proliferation, proangiogenetic pathways, and antitumoral immune response (5).This study evaluated the prognosis and expression of GBM patients by analyzing the mutation spectrum of PTEN-related microenvironment.

We collected a set of genes related to GBM. Candidate genes were obtained by differentially expressed genes. One-way Cox and Kaplan–Meier analyses were performed to identify the genes. Combined with LASSO regression analysis to establish prognostic features and further identify prognosis-related genes, a survival prognostic model was constructed, and the area under the ROC curve suggested that the model had high accuracy. Combined with nomogram analysis, the prognostic significance of genetic features in GBM was assessed.

## Materials and method

### Data acquisition

Expression matrices of all tumors, including the clinically relevant pathological features of tumor tissue samples, were obtained from the Cancer Genome Atlas (TCGA) database and evaluated based on the data completeness of clinical samples and degree of matching with sequenced samples. Duplicate and censored samples and cases without clinical findings were excluded.

### Variance analysis

The Limma package of R software can screen for differentially expressed genes between GBM and para cancer with |logFC| = 0.3785 (adj. *P*-value <0.05) and plotted volcanoes.To further confirm the potential functions of potential targets,the data were analyzed by functional enrichment. ClusterProfiler program package in R software was used to analyze the GO Function of potential mRNA and KEGG pathway gene ontology (GO) containing Molecular functions (MF), Biological process (BP) and Cellular Component (CC).

### Kaplan–Meier survival analysis

The effect of genetic characteristics on prognosis was verified by One-way Cox. Kaplan–Meier survival curves were plotted to compare the survival of patients in the high and low expression level groups.

### Establishment and analysis of risk prediction models

LASSO regression analysis was applied to narrow the range of prognosis-related genes and ensure the stability of the results. The median risk score of each sample was calculated using the risk score formula as the threshold, and patients were classified into high expression level and low expression level groups according to the expression level of PTEN. Survival curves were plotted. In addition, the working curves (ROC) of the subjects were plotted, and the area under the curve (AUC) was calculated to assess the predictive validity of the model.

### Nomogram analysis

Independent prognostic factors for GBM were determined by univariate and multifactorial analyses. Column plots and calibration curve plots predicted the predictive power of survival at 1, 2, and 3 years.

### Pan-cancer analysis

Data from normal and TCGA tumor tissues in the GTEx database were combined to analyze the differences in their gene expression. The amount of tumor mutations in each tumor sample was counted separately, and the relationship between gene expression and tumor mutational burden was analyzed.

## Results

### Mutation landscape

**Figure 1:**
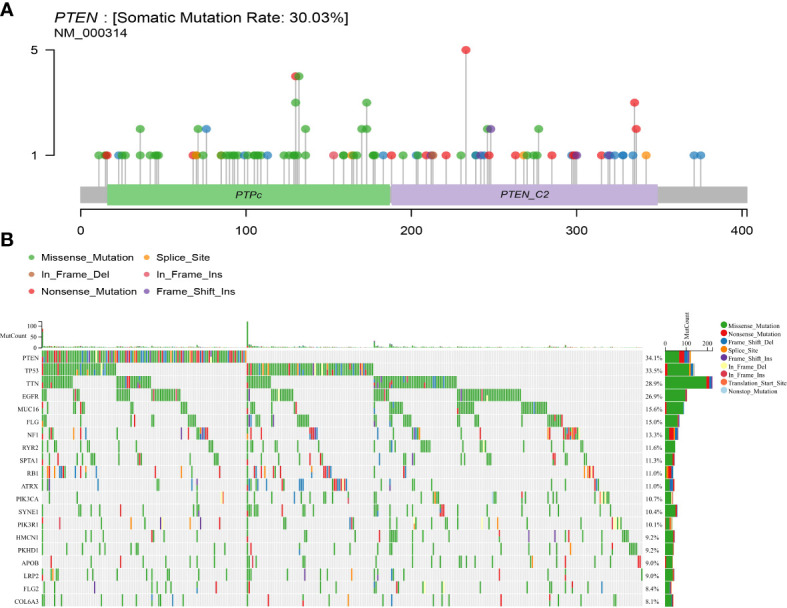
Somatic mutation rate and mutation landscape map of PTEN in GBM. **(A)** Lollipop plot of PTEN mutation distribution in GBM; **(B)** oncoplot showing the somatic landscape of the GBM cohort.

PTEN gene mutation data, clinical data, and transcriptome data from the TCGA data bank and somatic mutations in GBM patients were downloaded. Mutation analysis revealed that PTEN had a high mutation rate of 30.03% in GBM, which was the highest among TCGA tumors (**Figure 1A**). PTEN mutation frequency ranked first (34.1%) among all mutated genes in GBM (**Figure 1B**).

### Differentially expressed gene screening

**Figure 2:**
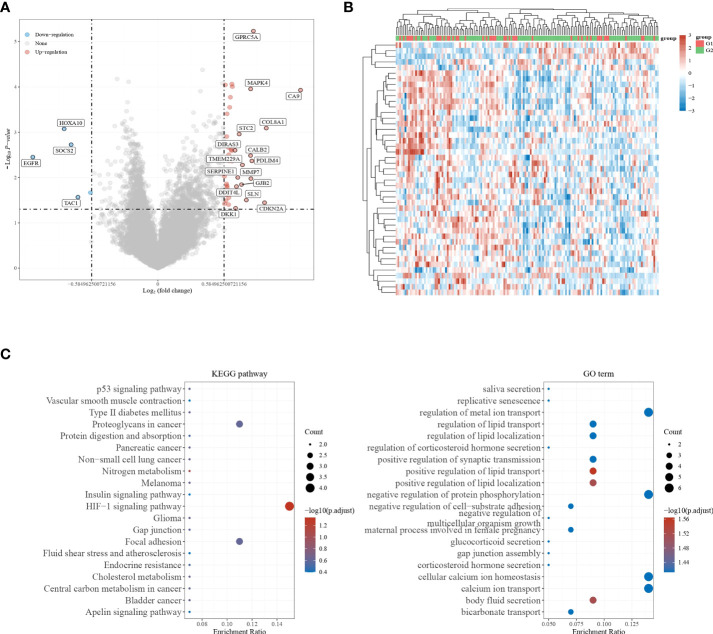
Screening for differentially expressed genes. **(A, B)** Volcano plot showing differentially expressed genes in GBM; **(C)** KEGG pathway enrichment results and GO term enrichment results.

In | logFC | = 0.58 under the condition of the filter, identified the genes from the TCGA - GBM data too little number, change | logFC | = 0.3785, identified 45 differentially expressed genes (adj. P-value <0.05). There were 5 down-regulated genes and 40 up-regulated genes (**Figures 2A, B**). The results of differentially up-regulated genes and down-regulated genes, KEGG pathway enrichment, and GO term enrichment are shown in **Figure 2C**.

### Prognostic analysis

**Figure 3:**
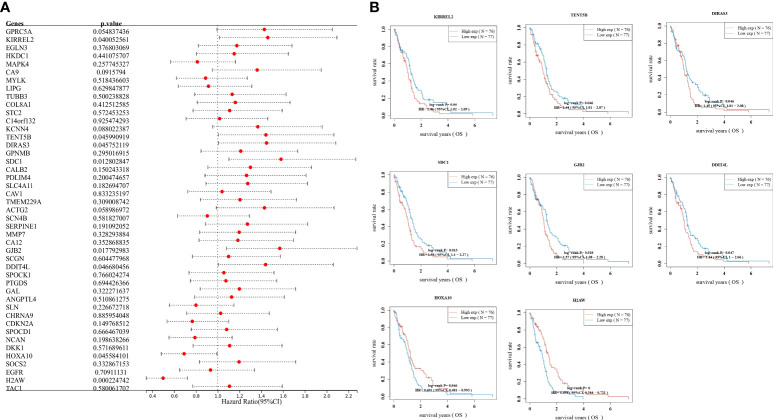
Prognostic analysis, **(A)** Validation of gene effects on prognosis using one-way Cox regression, presented using forest plots; **(B)** Kaplan–Meier survival analysis of the relationship between high and low expression levels of DDIT4L, DIRAS3, GJB2, H2AW, HOXA10, KIRREL2, SDC1, and TENT5B genes and GBM prognosis.

To understand the effect of each gene on prognosis, one-way Cox regression was used to validate the patients into high and low expression level groups by scoring each gene’s median expression level as the median value. It was further observed that only KIRREL2, TENT5B, DIRAS3, SDC1, GJB2, DDIT4L, HOXA10, and H2AW were prognostically significant in GBM (**Figure 3A**). The relationship between the prognosis of GBM and the expression levels of the above genes was further analyzed by plotting Kaplan–Meier survival curves. It was found that the higher the expression of DDIT4L, GJB2, KIRREL2, DIRAS3, HOXA10, SDC1, and TENT5B, the worse the prognosis, and the lower the expression of H2AW, the worse the prognosis (**Figure 3B**).

### Expression analysis

**Figure 4:**
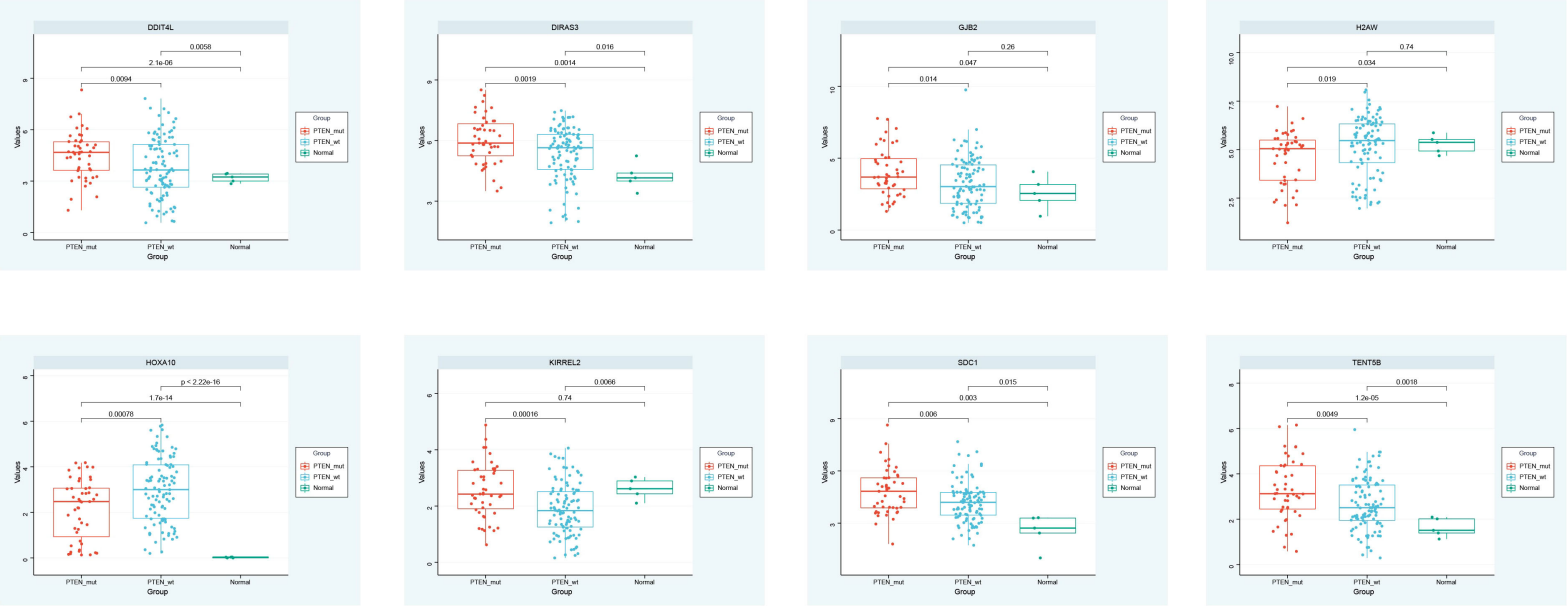
Expression analysis.

The expression of DDIT4L, DIRAS3, GJB2, H2AW, HOXA10, KIRREL2, SDC1, and TENT5B in the PTEN mutant group, PTEN wild group, and normal group were observed, and the box plot identified that DDIT4L, DIRAS3, GJB2, HOXA10, SDC1, and TENT5B were up-regulated in PTEN mutant group and PTEN wild group. H2AW and KIRREL2 were down-regulated in PTEN mutant group and PTEN wild group, compared with the normal group (**Figure 4**).

### Construction of the LASSO prognostic model

**Figure 5:**
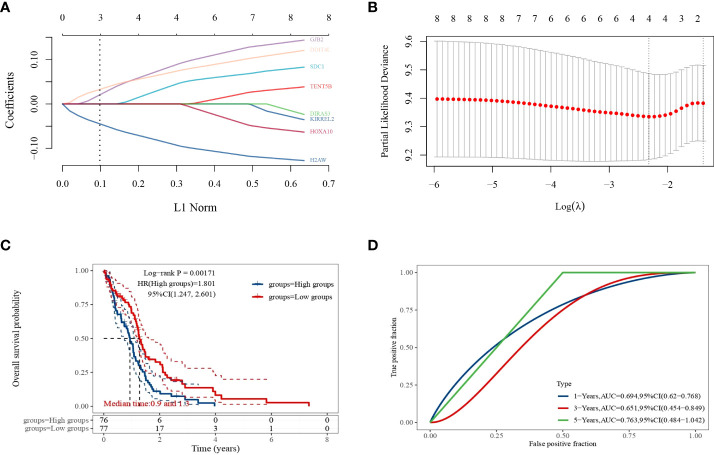
LASSO regression analysis. **(A)** Coefficients of selected features expressed as λ parameters; **(B)** partial likelihood deviation plotted with log(l) using LASSO regression analysis model; **(C)** Kaplan–Meier survival curves for patients in the high and low expression level groups; **(D)** time-dependent ROC curve plot.

LASSO regression analysis was performed to further narrow the range of prognosis-related genes and ensure the stability of the results (**Figures 5A, B**). The risk score was calculated for each sample using the following risk score formula:


lambda.min=0.0982

$$\begin{array}{l} \text{Riskscore}=\left(0.0267\right)\times \text{SDC}1+\left(0.0704\right)\times \text{GJB}2+\left(0.0625\right)\times \\ \text{DDIT}4\text{L}+\left(-0.0792\right)\times \text{H}2\text{AW} \end{array}$$

The median expression level score was used as a threshold for dividing the patients into high and low expression level groups and plotting the survival curves, which showed that the survival prognosis of the high expression level group was significantly worse than that of the low expression level group (**Figure 5C**). The ROC curves of 1, 3, and 5-year survival of GBM patients predicted by this risk score model were plotted (**Figure 5D**), and their AUC areas were 0.694,0.651, and 0.763, respectively.

### Nomogram analysis

**Figure 6:**
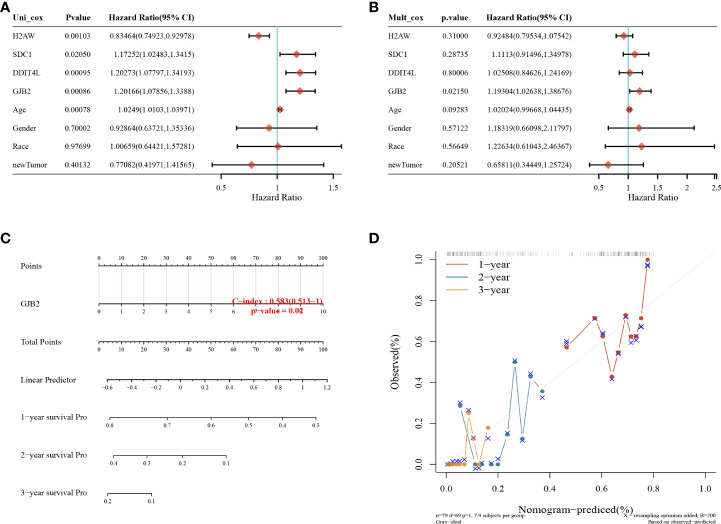
Nomogram analysis. **(A)** Univariate and **(B)** multifactorial Cox analyses showing that the GJB2 gene is an independent prognostic factor for GBM; **(C)** columnar plots predicting 1, 2, and 3-year overall survival in GBM patients; **(D)** calibration curves for the overall survival columnar plot model.

The GJB2 gene was shown to be a prognostic factor for GBM by univariate and multifactorial analyses (**Figures 6A, B**). Column and calibration curve plots indicated the predictive power of survival at 1, 2, and 3 years. The C-index was 0.583 and the *P*-value was 0.02.

### Expression of GJB2 in GBM

**Figure 7:**
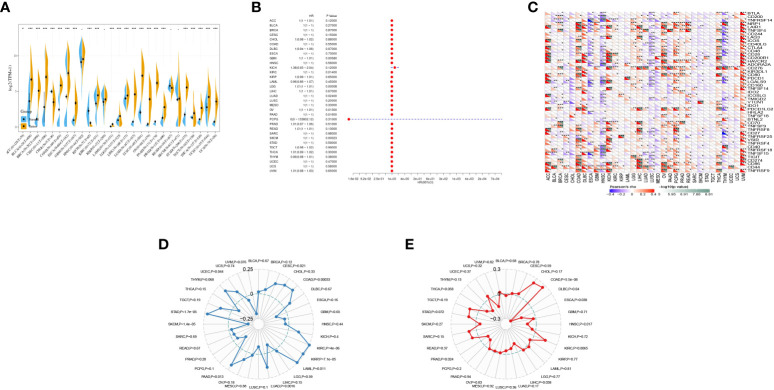
Pan-cancer analysis of GJB2 in multiple tumors. **(A)** Expression of GJB2 in multiple tumors; **(B)** prognostic analysis of GJB2 in multiple tumors; **(C)** correlation of GJB2 with immune checkpoints in multiple tumors; **(D)** correlation of GJB2 with immune mutation load in multiple tumors; **(E)** correlation of GJB2 with microsatellites in multiple tumors. *P<0.05, **P<0.01,***P<0.001.

The expression of GJB2 in tumors was analyzed by integrating the data from TCGA, and the results showed that GJB2 was highly expressed in BLCA, BRCA, CESC, COAD, DLBC, ESCA, GBM, HNSC, KIRC, KIRP, LGG, LIHC, LUAD, LUSC, OV, PAAD, PRAD, STAD, TGCT, THCA, UCEC, and UCS, and there was low expression in CHOL, KICH, and READ (**Figure 7A**).

One-way Cox regression showed prognostic significance of GJB2 in KICH and PCPG (**Figure 7B**).

The relationship between GJB2 gene expression and immune checkpoint gene expression was analyzed, and CD276 was highly correlated with multiple cancers (**Figure 7C**).

Immune mutation load correlation indicated that GJB2 was significantly associated with immune mutation load in CESC, COAD, KIRC, KIRP, LAML, LUAD, PAAD, SKCM, STAD, and UCEC (**Figure 7D**).

GJB2 was correlated with microsatellite instability in COAD, DLBC, ESCA, HNSC, KIRC, LIHC, and PRAD (**Figure 7E**).

## Discussion

As contemporary living standards and the material conditions of people continue to improve, health issues are receiving more and more attention. However, the incidence of cancer is increasing year by year, threatening human health. Among the cancers, GBM, which is classified as a grade IV diffuse glioma, is the primary tumor in adults and has a terrible prognosis. It has a high rate of recurrence and can spread rapidly to other parts of the brain (6), causing thousands of deaths worldwide each year. GBM consists of highly malignant cells that have metastatic and angiogenic properties that lead to resistance to agents such as temozolomide (3). The average survival of GBM patients remains below 20 months using current therapies (7), and conventional systemic chemotherapeutic agents used to treat GBM are ineffective. Several studies have found that the extremely high recurrence rate of GBM is associated with the expression of strongly proliferative genes in cells (8). Since this process usually involves multiple genes (9), we aimed to explore the prognostic genes that may play a role in the treatment of GBM.

PTEN, one of the most frequently mutated genes in human cancers (10), is a tumor suppressor with growth and survival regulatory functions (11). It controls many processes, including survival, proliferation, differentiation, energy metabolism, and deregulation of cell structure and mobility (12, 13). Deletion or mutation of PTEN leads to increased cell proliferation and decreased cell death and tumor development (14). Somatic loss-of-function mutations in PTEN can cause or drive the malignant phenotype of various human cancers (13), which is consistent with our findings. GJB2 is considered an oncogene and is related to tumor growth, EMT, and lymph node metastasis in a variety of cancers (15–18). Mutations in the GJB2 gene are a major cause of autosomal recessive hereditary nonsyndromic hearing loss (ARNSHL) in many populations (19). GJB2 expression is elevated in many tumor cell lines, tumor tissues (20), and breast cancer (21). It has been shown that GJB2 is an independent prognostic biomarker for LUAD, and patients with GJB2 overexpression have shorter overall survival. GJB2 may also be a potential prognostic factor for KIRC, as shown by pan-cancer analysis (22).

We identified genes related to the prognosis of GBM, namely GJB2. We first screened differentially expressed genes by differential analysis and performed one-way Cox regression and Kaplan–Meier analyses to identify genes related to prognosis. The groups were classified into high and low expression level, based on PTEN expression. Combined with LASSO regression analysis to establish prognostic characteristics and further identify prognosis-related genes, multi-factor Cox regression analysis was used to establish risk score equations for the prognostic model to construct the survival prognostic model, and the area under the ROC curve proved that the model had high accuracy. Combined with nomogram analysis, GJB2 was considered an independent predictor of GBM prognosis.

Many studies on the genetic correlation of GBM prognosis have been conducted. For example, Yanxin Li et al. found that the prognosis of GBM patients was poorer when the HOXD10 gene was highly expressed, and HOXD10 may play different roles at different stages of GBM development (23). To the best of our knowledge, the GJB2 gene that we screened is a new GBM biomarker, and no previous reports of this gene associated with the development and progression of GBM have appeared.

In summary, this study provides a predictive biological marker for GBM patients and confirms GJB2 as a key gene for GBM progression. These conclusions may provide a direction for prognosis prediction and treatment of GBM patients.

## Data availability statement

The original contributions presented in the study are included in the article/supplementary material. Further inquiries can be directed to the corresponding author.

## Funding

This study was supported by the grants from the Ningbo medical and health brand discipline (PPXK2018-04) and Ningbo Science and Technology Innovation 2025 Major Project (2020Z094, 2022Z125), Key Laboratory of Precision Medicine for Atherosclerotic Diseases of Zhejiang Province (2022E10026).

## Author contributions

We contributed equally for this work.

## Conflict of interest

The authors declare that the research was conducted in the absence of any commercial or financial relationships that could be construed as a potential conflict of interest.

## Publisher’s note

All claims expressed in this article are solely those of the authors and do not necessarily represent those of their affiliated organizations, or those of the publisher, the editors and the reviewers. Any product that may be evaluated in this article, or claim that may be made by its manufacturer, is not guaranteed or endorsed by the publisher.
